# When One Gland Speaks First: Autoimmune Polyendocrinopathy Syndrome Type 1 (APS-1) Unmasked by Isolated Hypoparathyroidism

**DOI:** 10.7759/cureus.102058

**Published:** 2026-01-22

**Authors:** Manal Mustafa, Abdullah Sabsabee, Mohamad Sabsabee

**Affiliations:** 1 Pediatric Endocrinology, Al Jalila Children’s Specialty Hospital, Dubai, ARE; 2 Pediatrics, University of Kalamoon, Kalamoon, SYR; 3 Pediatrics, Al Jalila Children’s Specialty Hospital, Dubai, ARE

**Keywords:** aire gene mutation, autoimmune polyendocrinopathy syndrome type 1, chronic mucocutaneous candidiasis, hypoparathyroidism, isolated hypoparathyroidism

## Abstract

Autoimmune polyendocrinopathy syndrome type 1 (APS-1) is a rare autosomal recessive disorder caused by pathogenic variants in the *AIRE* gene and classically characterized by the triad of hypoparathyroidism, chronic mucocutaneous candidiasis, and adrenal insufficiency. Although hypoparathyroidism is often the earliest manifestation, isolated and prolonged monosymptomatic presentations remain uncommon and may delay recognition of the syndrome. We report the case of a child who was first presented at four years of age with severe hypocalcemia in the setting of acute gastroenteritis and was diagnosed with hypoparathyroidism. Apart from hypomagnesemia and hyperphosphatemia, extensive workup revealed no additional autoimmune or endocrine abnormalities. Genetic testing subsequently identified a homozygous likely pathogenic stop-loss *AIRE* gene mutation, confirming APS-1. Over a three-year follow-up period, the patient remained clinically stable with well-controlled hypoparathyroidism, except for hypocalcemic episodes during diarrheal illnesses, and persistently normal adrenal, thyroid, pancreatic, and celiac screening. The first additional disease feature emerged three years after the initial presentation, at the age of seven years, when a fungal nail infection consistent with mucocutaneous candidiasis was noted. This case highlights the marked phenotypic variability with delayed evolution of APS-1 and underscores that isolated hypoparathyroidism, even when severe, may precede other disease components by several years. Early genetic testing in children with apparently isolated hypoparathyroidism allows anticipatory guidance, structured surveillance, and timely recognition of evolving autoimmune manifestations.

## Introduction

Autoimmune polyendocrinopathy syndrome type 1 (APS-1), also known as autoimmune polyendocrinopathy-candidiasis-ectodermal dystrophy, is a rare (prevalence of 1 per 90,000-200,000 individuals) autosomal recessive disorder caused by pathogenic variants in the *AIRE* gene, which plays a critical role in central immune tolerance [1,2]. Loss of *AIRE* function leads to defective negative selection of autoreactive T lymphocytes and predisposes affected individuals to a broad spectrum of organ-specific autoimmune diseases [1].

Clinically, APS-1 is classically defined by the presence of at least two components of the triad of chronic mucocutaneous candidiasis (CMC), hypoparathyroidism, and primary adrenal insufficiency [2,3]. The manifestations typically emerge sequentially during childhood. CMC most often represents the earliest clinical feature, usually appearing in the first few years of life. Hypoparathyroidism tends to develop later, most commonly in mid-childhood around the age of five years, with a slightly earlier onset reported in females. Primary adrenal insufficiency is generally a later manifestation, frequently arising around five years after hypoparathyroidism, during late childhood or adolescence [3-6].

In addition to the classic triad, APS-1 is associated with a wide range of autoimmune and ectodermal manifestations, including autoimmune thyroid disease, type 1 diabetes, autoimmune hepatitis, pernicious anemia, gonadal failure, alopecia, vitiligo, keratoconjunctivitis, pneumonitis, and tubulointerstitial nephritis [5-7]. Considerable inter- and intrafamilial variability has been well documented, with substantial heterogeneity in age of onset, sequence of manifestations, and disease severity, even among individuals carrying identical *AIRE* mutations [6,8].

Although hypoparathyroidism is a recognized component of APS-1, its presentation as a prolonged isolated manifestation in childhood remains uncommon and poses diagnostic challenges, particularly in the absence of early mucocutaneous candidiasis or adrenal involvement [4,8]. Documentation of such cases is important to illustrate the spectrum of APS-1 presentation, emphasize the limitations of relying solely on clinical criteria early in the disease course, and reinforce the role of genetic testing in establishing an early diagnosis. Reporting this case contributes to the existing literature by highlighting the delayed phenotypic evolution of APS-1 in a pediatric patient and underscores the need for ongoing surveillance and family counseling even when only a single disease component is initially apparent [8-10].

## Case presentation

A previously healthy four-year-old girl first presented in March 2022 with acute gastroenteritis characterized by diarrhea, abdominal pain, and vomiting complicated by seizure activity. During evaluation, she was found to have significant hypocalcemia and was treated with intravenous calcium. Further evaluation revealed hypomagnesemia, hyperphosphatemia, low parathyroid hormone levels, and low vitamin D levels. A diagnosis of hypoparathyroidism was made. The patient was commenced on alphacalcidol (active form of vitamin D), vitamin D3, and oral calcium supplement, and was referred to our endocrinology department.

At her initial follow-up pediatric endocrinology assessment, she was clinically well and asymptomatic; however, laboratory evaluation demonstrated persistently low ionized calcium levels (0.75 mmol/L, normal: 1.15-1.29 mmol/L). She continued calcium and active vitamin D therapy with close biochemical monitoring. Over subsequent follow-up visits, ionized calcium levels gradually improved, as shown in Table 1, allowing for progressive tapering and eventual discontinuation of oral calcium supplementation, while maintenance therapy with alphacalcidol and vitamin D3 was continued. The bone profile test results trend is presented in Table 2.

**Table 1:** Capillary ionized calcium trend.

Test (normal range)	Initial encounter	1 week follow-up	2 weeks follow-up	3 weeks follow-up
Capillary ionized calcium (1.15–1.29 mmol/L)	0.75 mmol/L	1.06 mmol/L	1 mmol/L	1.18 mmol/L

**Table 2:** Bone profile testing results trend.

Test (normal range)	Initial encounter	3 months follow-up	6 months follow-up	9 months follow-up	12 months follow-up
Total calcium (9.3–10.6 mg/dL)	6.5 mg/dL	7.6 mg/dL	8.1 mg/dL	7.4 mg/dL	7.1 mg/dL
Vitamin D (30–100 ng/mL)	23.8 ng/mL	-	-	46.9 ng/mL	-
Parathyroid hormone (11.3–60 pg/mL)	5.6 pg/mL	-	-	-	-
Phosphate (4–5.6 mg/dL)	9.6 mg/dL	6.4 mg/dL	7.3 mg/dL	7.1 mg/dL	6.9 mg/dL
Magnesium (1.7–2.1 mg/dL)	1.9 mg/dL	-	1.88 mg/dL	1.7 mg/dL	1.8 mg/dL

Given the early onset of hypoparathyroidism, genetic testing was performed and identified a homozygous likely pathogenic stop-loss *AIRE* gene mutation, as shown in Table 3, confirming the diagnosis of APS-1. Systematic screening for additional autoimmune and endocrine manifestations, including adrenal, thyroid, pancreatic, and gastrointestinal involvement, remained normal during serial follow-up. A short Synacthen test demonstrated an adequate cortisol response, and adrenal autoantibodies were negative.

**Table 3:** Genetic testing result detailing AIRE gene mutation.

Gene	Variant	Zygosity	Disease	Inheritance	Classification
*AIRE* (NM_000383.4)	c.1637G>C, p.(*546Serext*60)	Homozygous	Autoimmune polyendocrinopathy syndrome type 1	Autosomal dominant/Autosomal recessive	Likely pathogenic

**Figure 1:**
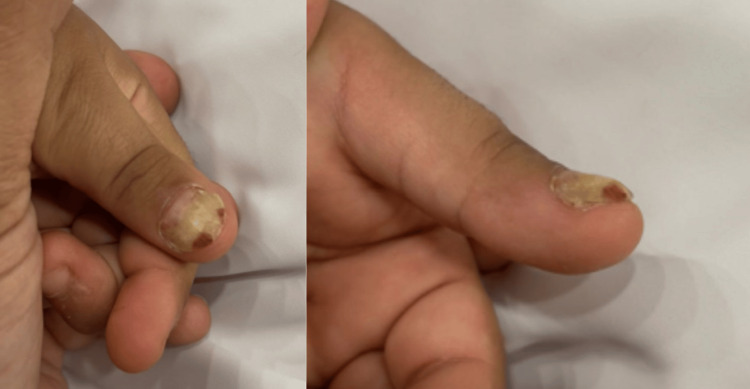
Fungal infection of the left thumbnail.

The patient remained clinically stable with hypoparathyroidism being the sole disease manifestation for approximately three years following the initial presentation. During this period, she would require calcium supplementation and an increased alphacalcidol dose during diarrheal illnesses, which can be tapered down after recovery. At the age of seven years, she developed a fungal infection involving the left thumbnail, as shown in Figure 1, which was treated with topical antifungal therapy and represented the first additional clinical feature consistent with mild mucocutaneous candidiasis. No other cutaneous, mucosal, or systemic autoimmune manifestations were identified.

Ongoing surveillance demonstrated normal thyroid function with negative thyroid antibodies, normal glycemic control with negative pancreatic autoantibodies, negative celiac screening, and preserved adrenal function. Annual renal ultrasonography remained normal, although a mildly elevated urine calcium-to-creatinine ratio of 0.32 mg/mg creatinine (normal value <0.2) was noted during follow-up. The patient continues treatment with alphacalcidol and vitamin D supplementation, maintaining stable calcium control under regular surveillance.

Her parents are not consanguineous, and family screening revealed a younger sibling who underwent genetic testing at 18 months of age and was found to carry the same homozygous pathogenic *AIRE* mutation. The sibling who is currently four years old remains clinically asymptomatic with normal biochemical screening to date.

## Discussion

The clinical expression of APS-1 unfolds over time and varies considerably between individuals. Manifestations related to mucocutaneous candidiasis often appear during the earliest years of life, whereas endocrine involvement tends to develop later. Hypoparathyroidism commonly emerges during mid-childhood and has been reported to occur at a younger age in female patients. In contrast, primary adrenal insufficiency is typically a later feature of the disease, frequently arising several years after the onset of hypoparathyroidism [3-6].

The reported case deviates from this typical pattern, with hypoparathyroidism as the initial and isolated manifestation, persisting for approximately three years before the development of nail-limited candidiasis. While hypoparathyroidism is a common component of APS-1, its occurrence as the sole presenting feature, particularly in the absence of candidiasis during early childhood, is less frequent and may delay recognition of the underlying syndrome [4,8].

The severity of the initial presentation, characterized by severe hypocalcemia complicated by seizures during an intercurrent illness, highlights that APS-1 may first manifest with life-threatening endocrine complications despite an otherwise limited phenotype. In such cases, absence of early CMC or adrenal involvement may lead to misclassification as idiopathic hypoparathyroidism. Increasing use of molecular diagnostics has demonstrated that a subset of children with apparently isolated hypoparathyroidism harbor pathogenic *AIRE* mutations, most commonly biallelic loss-of-function variants that disrupt central immune tolerance and underlie the autosomal recessive form of APS-1. Recognition of these mutations supports early genetic evaluation to enable timely diagnosis and anticipatory surveillance [8,9].

The delayed and subtle emergence of CMC in this patient further illustrates the heterogeneous and evolving nature of APS-1. Nail-only fungal infections represent a mild and often underrecognized form of candidiasis and may precede more extensive mucocutaneous involvement [5,7]. Reliance on overt mucosal candidiasis for diagnosis may therefore underestimate disease progression. Long-term follow-up is particularly critical given the risk of adrenal insufficiency, which may develop years after initial diagnosis and carries significant morbidity if unrecognized [3,6].

Ferreira et al. described a 50-year-old man with hypoparathyroidism diagnosed at age seven that remained the sole major manifestation for decades; subsequent evaluation later confirmed both CMC and Addison’s disease, resulting in delayed recognition of APS-1 [11]. Sahoo et al. reported three cases of APS-1 where one patient developed isolated hypoparathyroidism at 14 years of age and premature ovarian insufficiency 14 years later [12]. These reports, together with our case, emphasize that APS-1 may evolve slowly and that single-component presentations warrant long-term surveillance.

Finally, the identification of an asymptomatic sibling, currently four years old, with the same homozygous pathogenic *AIRE* mutation emphasizes the marked intrafamilial variability and age-dependent penetrance characteristic of APS-1. Even among individuals with identical genotypes, the timing, sequence, and severity of disease manifestations may differ substantially [6,8]. This observation underscores the importance of proactive biochemical monitoring and structured long-term surveillance in genetically affected but clinically asymptomatic individuals.

## Conclusions

This case demonstrates that APS-1 may present with hypoparathyroidism as the initial and isolated manifestation, despite CMC being the more typical first feature. Severe hypocalcemia may be the sole presenting manifestation and can precede other disease components by several years. Early molecular diagnosis facilitates appropriate family counseling and structured long-term surveillance, which are essential for timely detection and management of evolving autoimmune complications.
